# TRPS1: A Marker of Follicular Differentiation

**DOI:** 10.3390/dermatopathology10020025

**Published:** 2023-06-14

**Authors:** Kristin J. Rybski, Hatice B. Zengin, Bruce R. Smoller

**Affiliations:** Department of Pathology and Laboratory Medicine, University of Rochester Medical Center, Rochester, NY 14642, USA; hatice_zengin@urmc.rochester.edu (H.B.Z.); bruce_smoller@urmc.rochester.edu (B.R.S.)

**Keywords:** TRPS1, hair follicle, follicular differentiation, trichoblastoma, trichoepithelioma, basal cell carcinoma

## Abstract

The trichorhinophalangeal syndrome type 1 (TRPS1) immunohistochemical (IHC) stain has increased in use in recent years as a marker for breast carcinomas. The TRPS1 gene is involved in various tissues, including the growth and differentiation of hair follicles. This article seeks to evaluate the IHC expression of TRPS1 in cutaneous neoplasms with follicular differentiation, such as trichoblastoma (TB), trichoepithelioma (TE), and basal cell carcinoma (BCC). IHC studies were performed on 13 TBs, 15 TEs, and 15 BCCs with an antibody against TRPS1. The study found a variable staining expression of TRPS1 in the tumor nests of TB, TE, and BCC. BCCs were distinct in that none of the BCCs demonstrated intermediate or high positivity, while TBs and TEs showed intermediate-to-high positivity in 5/13 (38%) and 3/15 (20%) of cases, respectively. We observed a distinct staining pattern among the mesenchymal cells of TB and TE. We found that TRPS1 highlighted perifollicular mesenchymal cells adjacent to the nests of TB and TE tumor cells. This staining pattern was absent in BCCs, where only scattered stromal cells were positive for TRPS1. Papillary mesenchymal bodies were also highlighted by TRPS1 in TB and TE. TRPS1 stained various parts of the normal hair follicle, including the nuclei of cells in the germinal matrix, outer root sheaths, and hair papillae. TRPS1 may be a useful IHC marker for follicular differentiation.

## 1. Introduction

The use of the trichorhinophalangeal syndrome type 1 (TRPS1) immunohistochemical stain has increased in routine clinical practice in recent years for its utility as a highly sensitive and specific marker for breast carcinomas, including triple-negative breast carcinomas [1]. Mutations in the gene encoding for the TRPS1 GATA zinc finger transcription factor result in trichorhinophalangeal syndrome type I and type III, which are autosomal-dominant disorders characterized by fine and sparse scalp hair, craniofacial abnormalities, and skeletal defects [2,3]. The TRPS1 gene is involved in a variety of tissues, including the development and differentiation of hair follicles [4]. The TRPS1 transcription factor has been shown to regulate epithelial proliferation during the development and differentiation of hair follicles and is expressed in the dermal papillae and mesenchymal cells surrounding the hair pegs [5,6]. Recently, a study demonstrated that many benign and malignant sweat gland tumors also show TRPS1 positivity [7]. The expression of TRPS1 in benign and malignant cutaneous neoplasms with follicular differentiation is currently unknown. Differential staining of TRPS1 may be utilized as a marker of follicular morphogenesis, such as in trichoblastoma (TB), trichoepithelioma (TE), and basal cell carcinoma (BCC)—cutaneous neoplasms with follicular differentiation. We hypothesized that TB, TE, and BCC may represent a continuum of follicular differentiation and anticipated that trichoblastomas and trichoepitheliomas would exhibit a distinct staining pattern of TRPS1, which would differ from that of basal cell carcinomas.

## 2. Materials and Methods

This study was a retrospective analysis using archival tissue. Our institution’s pathology database was used to query cases with the diagnosis of trichoblastoma, trichoepithelioma, and nodular-type basal cell carcinoma between the dates of 1 January 2005 and 31 December 2018. This was a monocentric study using cases signed out by our institution’s pathologists. Unstained, formalin-fixed, and paraffin-embedded sections were cut from archived tissue blocks of these specimens.

Immunohistochemical (IHC) studies were performed with an antibody against TRPS1 (clone EPR16171 from Abcam) on 4 µm sections of formalin-fixed, paraffin-embedded tissues from 13 trichoblastomas, 15 trichoepitheliomas, and 15 basal cell carcinomas (nodular type). The slides were reviewed to confirm each diagnosis. In terms of criteria considered in the selection of cases, trichoblastoma and trichoepithelioma were best thought of as a spectrum, with trichoblastoma being the least differentiated. Various morphological criteria were used to distinguish between TE and TB, including that TBs tended to be dermal tumors with poorly differentiated basaloid cells, while TEs were more superficial, often with connections to the epidermis, had more differentiated tumor cells, and more commonly had horn cysts. Characteristics common to both TE and TB that differentiated them from basal cell carcinoma were papillary mesenchymal bodies and cellular stroma directly adjacent to tumor nests. The Leica Bond III instrument was used for immunohistochemical studies. Antigen retrieval was performed with Bond Solution #2 (pH 9.0). The TRPS1 antibody (1:6000 dilution) was incubated for 8 min at room temperature. Tissue staining was performed on a Leica BOND III immunostainer with a Leica Refine Polymer Detection Kit. The breast carcinoma HER2 control kit served as an external positive control for TRPS1.

**Figure 1:**
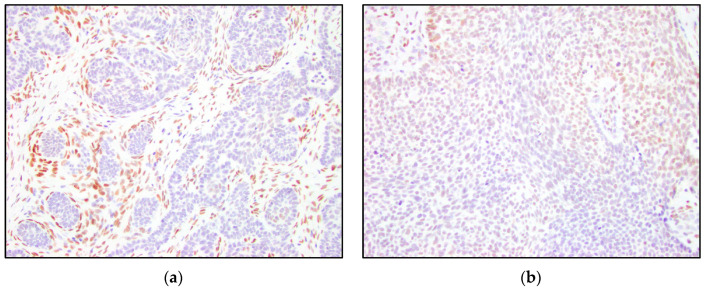

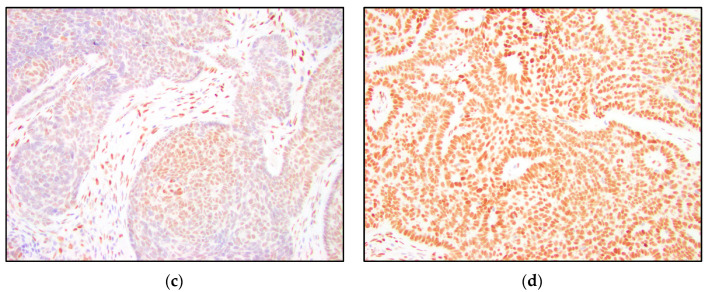
Examples of TRPS1 immunoreactivity scores in the tumor nests. (**a**) Negative TRPS1 score in trichoepithelioma and positive staining of mesenchymal cells (200×); (**b**) low positive TRPS1 score in basal cell carcinoma (200×); (**c**) intermediate positive TRPS1 score in trichoepithelioma (200×); (**d**) high positive TRPS1 score in trichoblastoma (200×).

The frequency, pattern, intensity, and proportion of expression in the tumor cells were then evaluated. The immunoreactivity scores for TRPS1 were calculated by multiplying the percentage of positive cells, represented as a whole number between 0 and 3 (0 ≦1%, 1 = 1%–10%, 2 = 11%–50%, and 3 = 51%–100%), by the number representing the staining intensity (0 = negative, 1 = weak, 2 = moderate, and 3 = strong). The immunoreactivity scores were considered negative (0–1), low positive (2), intermediate positive (3–4), or high positive (6 and 9) for TRPS1 (Figure 1). This scoring system was based on the previous study demonstrating TRPS1 immunohistochemistry staining as a marker for breast cancer [1].

## 3. Results

There was a variable IHC expression of TRPS1 in the epithelial components of TB, TE, and BCC. TRPS1 IHC demonstrated positivity in the tumor nests in six of thirteen (46%) trichoblastomas, nine of fifteen (60%) trichoepitheliomas, and five of fifteen (33%) nodular basal cell carcinomas. While TB and TE more often stained positively with TRPS1, many BCCs were also weakly positive, and overall, the staining within the tumor nests was inconsistent. Although TRPS1 staining was variable, BCCs were distinct in that none of the BCCs demonstrated intermediate or high positivity. On the other hand, five of thirteen (38%) TBs and three of fifteen (20%) TEs showed intermediate-to-high positivity, while zero of fifteen (0%) BCCs showed intermediate-to-high TRPS1 positivity (Table 1). A patchy staining pattern was noted in the majority of cases (Figure 1a–c), except for the cases with a high positive TRPS1 immunoreactivity score, which demonstrated a diffuse staining pattern (Figure 1d).

**Figure 2:**
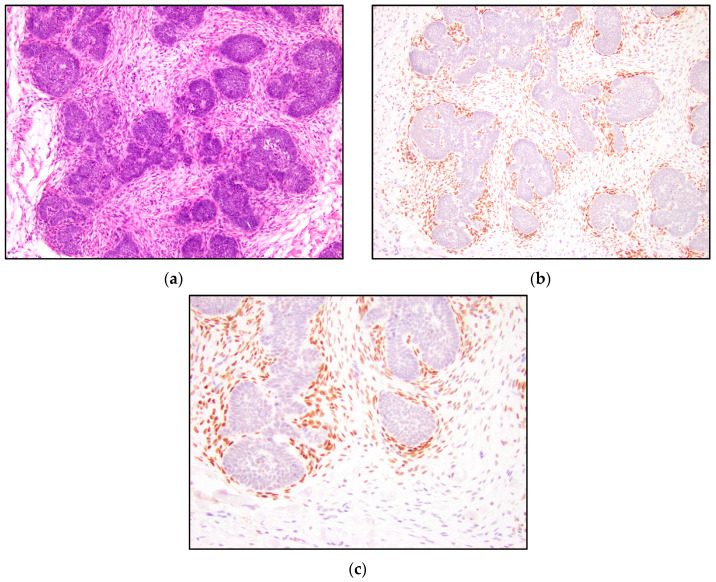
TRPS1 IHC highlighting perifollicular mesenchymal cells in trichoblastoma. (**a**) Trichoblastoma (H&E, 100×); (**b**,**c**) TRPS1 strongly stained mesenchymal cells surrounding the tumor nests in trichoblastoma (100× and 200×).

**Figure 3:**
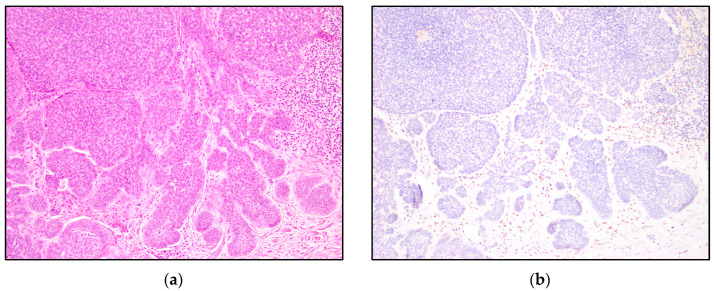

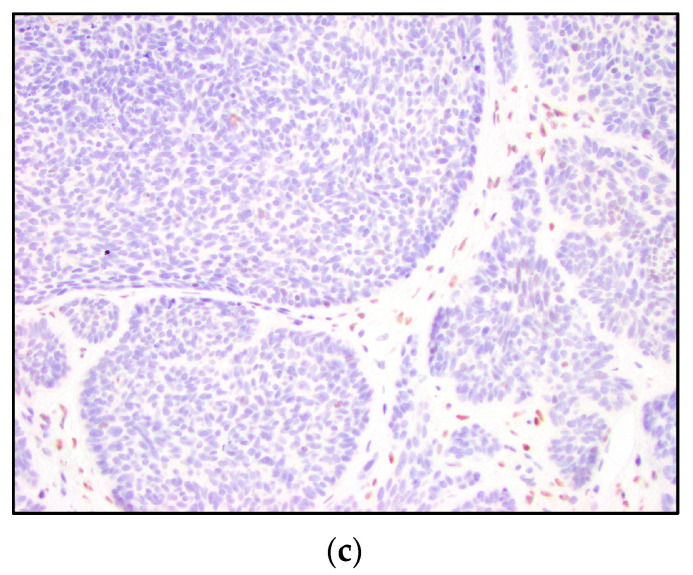
TRPS1 IHC demonstrating scattered mesenchymal cells in basal cell carcinoma. (**a**) Basal cell carcinoma (H&E, 100×); (**b**,**c**) TRPS1 stains scattered mesenchymal cells in basal cell carcinoma (100× and 200×).

**Figure 4:**
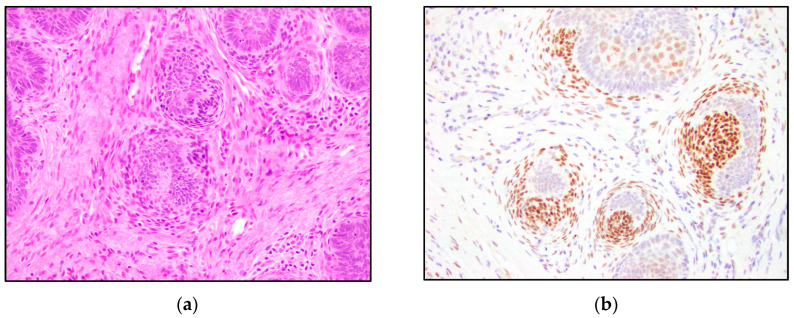
TRPS1 IHC staining papillary mesenchymal bodies in trichoepithelioma. (**a**,**b**) Trichoepithelioma with papillary mesenchymal bodies highlighted by the TRPS1 stain (H&E and TRPS1, 200×).

TRPS1 highlighted perifollicular mesenchymal cells adjacent to the nests of TB and TE tumor cells (Figure 2), while the uninvolved dermis was negative. This distinct staining of perifollicular cells was absent in basal cell carcinoma (Figure 3). The mesenchymal cells were clustered differently around the tumor nests of basal cell carcinoma, with only scattered stromal cells staining positive (Figure 3). Additionally, TRPS1 highlighted papillary mesenchymal bodies in TB and TE (Figure 4). Papillary mesenchymal bodies were not seen in any of the examined sections of BCC.

**Figure 5:**
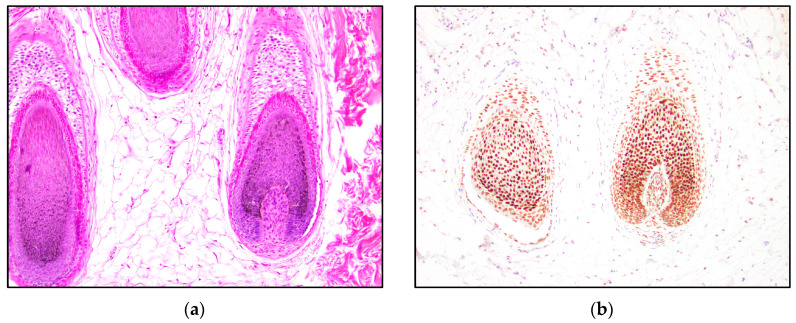
TRPS1 IHC expression in normal hair follicles. (**a**) Normal hair follicle (H&E, 100×); (**b**) TRPS1 staining the root sheaths, germinative cells, matrix cells, and dermal papilla of a hair follicle (100×).

In normal hair follicles, TRPS1 staining is seen in the outer root sheath, inner root sheath, follicular germinative cells, hair matrix cells, and the follicular dermal papilla (Figure 5). The staining intensity appears strongest at the hair bulb. Other skin components that stain positive for TRPS1 include the nuclei of sebaceous glands and eccrine glands. Keratinocytes are also often weakly positive for TRPS1.

## 4. Discussion

Trichoblastomas and trichoepitheliomas are benign cutaneous neoplasms with follicular differentiation, originating from germinative cells of the hair follicle. Sometimes, TBs and TEs are difficult to distinguish clinically and histologically from BCCs. Clinically, TE presents as a skin-colored to brown papule or nodule with a predilection for the head and neck. Basal cell carcinoma is the most common malignancy worldwide and a frequent diagnosis faced by dermatopathologists. It most commonly presents as a pearly, tan-pink nodule with overlying telangiectasias on sun-exposed areas of the skin. Trichoblastoma and trichoepithelioma may appear clinically similar to basal cell carcinoma [8]. Surgical biopsy is often performed to analyze histopathological differences to diagnose and differentiate these entities. Differentiating TB and TE from BCC can be challenging in cases, due to overlapping histological similarities [9,10]. The distinction is important to make because the prognoses and treatments differ. Histologically, basaloid epithelial cells are seen with prominent palisading at the periphery of the tumor. Papillary mesenchymal bodies, which represent abortive attempts to form the papillary mesenchyme, have been recognized as a histological feature in TE, useful in differentiating this entity from BCC [11]. Peripheral clefting, lymphocytic infiltrate, and myxoid stroma are features of BCC that have been noted to help distinguish BCC from other basaloid neoplasms [10].

Previous studies have attempted to differentiate these entities by utilizing various IHC stains. These include BCL-2, CD34, lectin peanut agglutinin (PNA), cytokeratin 15, cytokeratin 20 (CK20), CD10, D2-40, Ki67, p53, PCNA, and androgen receptor (AR) [12,13,14,15,16,17,18,19,20,21,22,23,24]. CK20, which stains Merkel cells within the tumor nests of trichoepitheliomas, is considered a useful immunohistochemical marker, as Merkel cells are generally not present in basal cell carcinomas [25,26]. CK20, however, has its limitations, as Merkel cells are scattered and may not be present in the representative sample in small specimens. PHLDA1 (Pleckstrin homology-like domain, family A, member 1), also known as TDAG51 (T-cell death-associated gene 51), is a follicular bulge stem cell marker that has been shown to be important and superior to CK20 in differentiating trichoepithelioma and basal cell carcinoma, especially in small skin biopsies [27,28].

**Figure 6:**
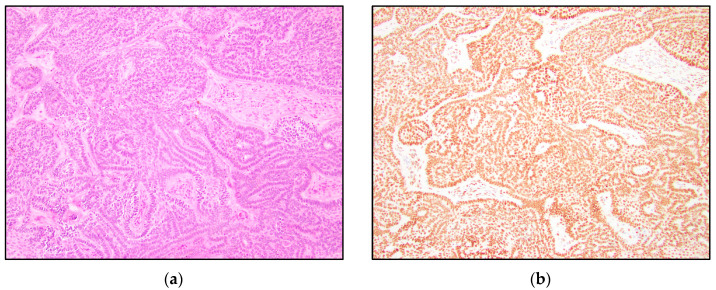
(**a**) Trichoblastoma (H&E, 100×). (**b**) High positive TRPS1 immunoreactivity in the trichoblastoma tumor nests (100×).

**Figure 7:**
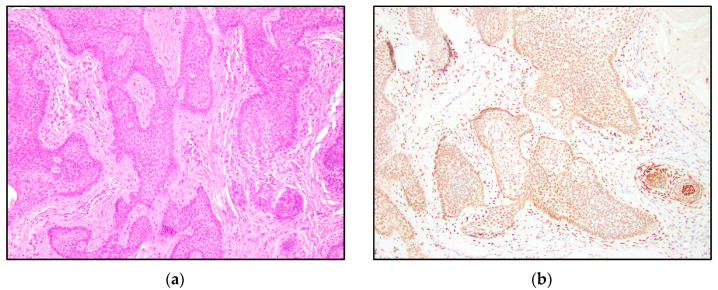
(**a**) Trichoepithelioma with a papillary mesenchymal body in the lower right corner (H&E, 100×). (**b**) High positive TRPS1 immunoreactivity in trichoepithelioma tumor nests and the papillary mesenchymal body (100×).

**Figure 8:**
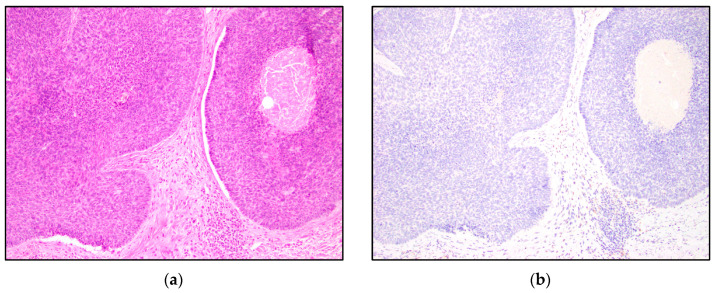
(**a**) Basal cell carcinoma with characteristic clefting (H&E, 100×). (**b**) Negative TRPS1 immunoreactivity in BCC tumor nests (100×).

Our study suggests that TRPS1 may provide only limited benefit to differentiating TB and TE from BCC, due to the variable staining patterns in the tumor nests and patchy staining patterns. The majority of low positive and intermediate positive cases demonstrate a patchy distribution of positive cells (Figure 1b,c). The patchy pattern of staining poses an important limitation in partial biopsy specimens where only a small part of the tumor is available for histologic evaluation. Therefore, it is imperative to note the possibility of false-negative staining in cases with limited diagnostic material. A high level of positive TRSP1 staining, however, may be useful in distinguishing these entities. High positivity and intermediate positivity were specific to TB and TE (Table 1, Figure 6 and Figure 7). BCC did not demonstrate any intermediate or high positive immunoreactivity (Table 1, Figure 8). The cases with high positivity showed diffuse staining patterns (Figure 1d, Figure 6 and Figure 7). Additionally, another strong differentiating feature between TB/TE and BCC was the staining pattern of the surrounding mesenchymal cells. TRPS1 highlighted a unique staining pattern among the mesenchymal cells surrounding tumor nests in TB and TE, appearing to recapitulate structures of the hair follicle.

We hypothesized that TB, TE, and BCC might represent a spectrum of follicular differentiation. We expected that a differentiating staining pattern would emerge in trichoblastomas and trichoepitheliomas, compared to basal cell carcinomas. While TRPS1 IHC staining within the tumor nests might not be especially helpful in distinguishing BCC from TB and TE, a characteristic stromal staining pattern was apparent. The stroma of TB and TE typically has a morphological appearance that simulates the stroma that surrounds normal hair follicles. The perifollicular mesenchymal cells immediately adjacent to the TB and TE tumor nests were highlighted by TRPS1 immunostaining (Figure 2). In contrast, none of the BCC cases were stained with this stromal pattern; TRPS1 simply demonstrated scattered mesenchymal cells in the stroma of the BCCs (Figure 3). In the cases of BCCs, clefts between the tumor lobules and stroma were also seen. Peritumoral clefting is a retraction artifact and a characteristic feature of BCC. The distinct stromal patterns may be due to TB and TE recapitulating the dermal papillae, which form from adjacent clusters of dermal mesenchymal cells and are involved in epithelial–mesenchymal interactions [29]. The epithelial–mesenchymal signals may be involved in the clustering of mesenchymal cells next to the epithelial tumor nests. TRPS1 stains perifollicular mesenchymal cells in a pattern similar to that previously shown with the CD34 IHC stain, which demonstrated a strong expression of CD34 in the mesenchymal cells directly adjacent to the epithelial tumor nests in TE [14]. In this prior study, it was suggested that the difference in staining patterns might be due to the presence of a different manner of tumor expansion and host response in TE, compared to BCC [14].

This study demonstrated consistent TRPS1 staining of perifollicular mesenchymal cells and papillary mesenchymal bodies in trichoblastomas and trichoepitheliomas. The staining pattern of TRPS1 in normal hair follicles demonstrated a similar pattern to that seen in trichoblastoma and trichoepithelioma. The staining of the dermal papillae in normal hair follicles (Figure 5) was similar to the staining of the stromal cells clustered around TB and TE tumor nests (Figure 2). Neoplasms derived from or differentiating towards the hair follicle represent a subgroup of cutaneous adnexal tumors. Trichoblastomas and trichoepitheliomas, as their first root word “tricho-” suggests, originate from cells of the hair follicle, specifically the germinative cells. The germinative cells and hair papillae in normal hair follicles stained positive for TRPS1 (Figure 5). Trichoblastomas and trichoepitheliomas are thought to originate from these structures. Cases of TB and TE that are further in the differentiation process may recapitulate the staining pattern of the normal hair follicle, indicating that TRPS1 staining may function as a marker of follicular differentiation.

Whether BCC is a tumor of epithelial or follicular differentiation has been debated in literature [30,31,32,33]. Many basal cell carcinomas arise de novo from undifferentiated stem cells and therefore have the capacity to differentiate into a wide range of directions, as stem cells do. Having this undifferentiated potential may represent one reason for the absent staining in many of the BCC cases. Traditionally, basal cell carcinoma is believed to arise from the basal layer of the epidermis; however, there has been evidence that BCC originates from the outer root sheath of the hair follicle [32,33]. TRPS1 stained various structures of the normal hair follicle, including the outer root sheath, the inner root sheath, the germinative cells, and the hair papillae (Figure 5). Although the epidermis sometimes showed TRPS1 positivity, the staining was underwhelming, compared to the staining of follicular components. The nuclei of the outer root sheath were more weakly positive than the germinative cells of the hair follicle. This might explain the low positive staining in many of the basal cell carcinomas and lend further evidence to a follicular origin in BCC.

On the other hand, the hair follicle is dynamic, with keratinocytes that are continuously growing and differentiating. The components of the hair follicle can change during the phases of the hair cycle, during which the structure of the hair follicle is altered. A previous study examined the immunohistochemical expression of cytokeratins and hair keratins differentially expressed in trichioblastomas and basal cell carcinomas. Both TB and BCC demonstrated widespread staining of a subset of cytokeratins characteristic for the outer root sheath suprabasal layer (CK14, CK17) and the companion layer (CK6hf) of the hair follicle [34]. TB, TE, and BCC might all simulate a variety of the components of a hair follicle. The variable staining we saw might suggest that TB, TE, and BCC do not recapitulate only one specific structure of the hair follicle but a variety of the follicular components. The staining of normal hair follicles and the similarities to the structures recapitulated by cutaneous follicular neoplasms highlight the potential use of TRPS1 as a marker of hair follicles and cells with follicular origin or differentiation.

TRPS1 is involved in follicular differentiation, and the TRPS1 IHC stain is positive in various components of the hair follicle; however, the precise role and IHC expression of TRPS1 in the hair follicle has not been fully established. A previous investigation reported that transplanted TRPS1-deficient skin in mice produced a substantial amount of hair, comparable to the wild-type, demonstrating that TRPS1 is not necessary for hair to develop [35]. While the absence of TRPS1 did not prevent hair growth, the transplanted TRPS1-deficient hair was softer, and the hair follicles and shafts were smaller in diameter. This suggests that TRPS1 plays a role in the growth and size of the hair follicle. Future studies exploring TRPS1 IHC immunoreactivity during the growth and differentiation of follicular structures may be informative.

## 5. Conclusions

The differential IHC staining among trichoblastomas/trichoepitheliomas and basal cell carcinomas, including highlighting perifollicular mesenchymal cells and papillary mesenchymal bodies in TB and TE, combined with the staining pattern in normal hair follicles, suggests that TRPS1 may be used as a marker of hair follicle differentiation. Although TRPS1 does not appear to definitively distinguish TB and TE from BCC, it may be a useful immunohistochemical marker for follicular differentiation and papillary mesenchymal bodies. Further studies will be necessary to explain the precise function of TRPS1 in hair morphogenesis and the utility of TRPS1 as a marker of follicular differentiation.

## Figures and Tables

**Table 1:** TRPS1 IHC immunoreactivity scores in the tumor nests of TB, TE, and BCC.

TRPS1 Expression	Trichoblastoma *N* = 13	Trichoepithelioma *N* = 15	Basal Cell Carcinoma *N* = 15
Negative *n* (%)	7 (54%)	6 (40%)	10 (67%)
Low Positive *n* (%)	1 (8%)	6 (40%)	5 (33%)
Intermediate Positive *n* (%)	2 (15%)	1 (7%)	0 (0%)
High Positive *n* (%)	3 (23%)	2 (13%)	0 (0%)

## Data Availability

Data is contained within the article.
